# Human Infection with West Nile Virus, Xinjiang, China, 2011

**DOI:** 10.3201/eid2008.131433

**Published:** 2014-08

**Authors:** Zhi Lu, Shi-Hong Fu, Lei Cao, Cheng-Jun Tang, Song Zhang, Zhao-Xia Li, Mamutijiang Tusong, Xin-Hua Yao, Hai-Lin Zhang, Pi-Yu Wang, Maimaitijiang Wumaier, Xue-Yan Yuan, Ming-Hua Li, Chang-Zhong Zhu, Li-Ping Fu, Guo-Dong Liang

**Affiliations:** State Key Laboratory for Infectious Disease Prevention and Control, Beijing, China (Z. Lu, S.-H. Fu, C.-J. Tang, M.-H. Li, G.-D. Liang);; National Institute for Viral Disease Control and Prevention, Beijing (Z. Lu, S.-H. Fu, C.-J. Tang, M.-H. Li, G.-D. Liang);; Collaborative Innovation Center for Diagnosis and Treatment of Infectious Diseases, Hangzhou, China (Z. Lu, S.-H. Fu, C.-J. Tang, M.-H. Li, G.-D. Liang);; Chinese Academy of Sciences, Beijing (L. Cao);; Xinjiang Center for Disease Control and Prevention, Urumqi, China (S. Zhang, M. Wumaier, L.-P. Fu);; Kashi Center for Disease Control and Prevention, Kashi, China (Z.-X. Li, C.-Z. Zhu);; Jiashi County Center for Disease Control and Prevention, Jiashi, China (M. Tusong, X-H Yao);; Yunnan Institute of Endemic Disease Control and Prevention, Dali City, China (H.-L. Zhang, P.-Y. Wang);; Jiashi County Hospital, Jiashi (X.-Y. Yuan)

**Keywords:** West Nile virus, viruses, human infection, encephalitis, Xinjiang, China

**To the Editor:** West Nile virus (WNV) is a mosquito-borne flavivirus in the Japanese encephalitis serocomplex of the family *Flaviviridae* (*1*). It has been reported in Africa, Asia, Europe, Australia, and North America, and is recognized as the most globally widespread mosquito-borne flavivirus (*2*). Isolation of WNV has previously been attempted in China, Japan and South Korea; however, no virus has been isolated (*3*–*5*). We report isolation of WNVs from mosquitoes in Xinjiang Uyghur Autonomous Region in western China. We also provide evidence of WNV human infections confirmed by IgM ELISA and seroconversion by 90% plaque reduction neutralization tests of paired serum samples obtained from persons with febrile illness and viral encephalitis in 2011.

Arbovirus surveillance was performed in the Kashi Region, Xinjiang, China, in August 2011. Mosquitoes were captured by using light traps and gravid traps for 15 days (days 1–5, 11­–15, and 21–25) at 9 collection sites in 9 villages in 2 townships. A total of 7,122 mosquitoes, representing 3 genera and 7 species in 118 pools, were tested. Mosquitoes collected were *Culex pipiens pipiens* (65.0%, 4,629/7,122), *Aedes flavidorsalis* (24.1%, 1,717/7,122), *Ae. caspius* (10.1%, 716/7,122), and other *Aedes* and *Culex* species (0.8%, 40/7,122) mosquitoes.

Mosquitoes were homogenized, and viral RNA was extracted directly from mosquito pools and amplified by using PCR and primers specific for WNV envelope (E) and nonstructural protein 5 genes as described (*6*). A total of 12 pools of *Cx. p. pipiens* mosquitoes were positive for WNV, which was confirmed by nucleotide sequencing. The minimum infection rate for *Cx. p. pipiens* mosquitoes was 2.56 infections/1,000 specimens tested.

In addition, supernatants of the 12 WNV-positive mosquito pools were inoculated onto Vero cells. Five pools yielded 5 virus isolates designated XJ11129–3, XJ11138–6, XJ11141–4, XJ11146–4, and XJ11148–2. The Vero cells aggregated and began shedding virus by 72 h postinfection.

Phylogenetic comparisons of complete nucleotide sequences of E gene from the 5 Xinjiang isolates (Figure, panel A) showed a high degree of genetic identity of lineage 1 with other highly pathogenic WNV strains, such as WNV NY99 and isolates from Russia. Nucleotide and amino acid sequences showed ≥99% identity with isolates from Russia (1999–2004) (*7*).

**Figure:**
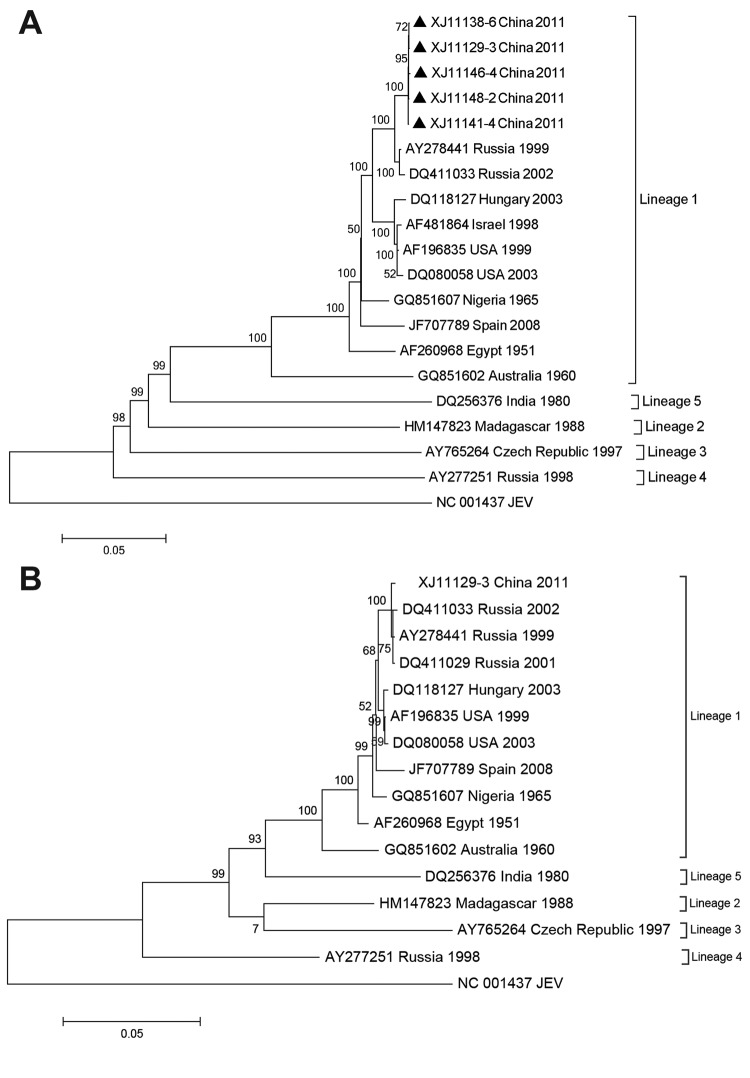
Phylogenetic analyses of A) envelope gene nucleotide sequence from 5 West Nile virus isolates (black triangles) from Xianjiang, Uyghur Autonomous Region, China, 2011, and B) nucleotide sequence of complete coding region of 1 isolate from Xinjiang (XJ11129–3). Trees were constructed by using MEGA 5.05 (http://www.megasoftware.net/) and maximum-likelihood with Kimura 2-parameter model parameter distances. Bootstrap values along branches are for 1,000 replicates. Trees were rooted by using Japanese encephalitis virus (JEV) as the outgroup virus. USA, United States. Scale bars indicate nucleotide substitutions per site.

The complete nucleotide sequence of XJ11129–3 contained 11,029 nt, and the phylogenetic tree of the nucleotide coding region showed similar topology with the E gene tree (Figure, panel B). Nucleotide sequences of E genes from XJ11138–6, XJ11141–4, XJ11146–4, and XJ11148–2 and the complete genome sequence of XJ11129–3 were submitted to GenBank under accession nos. JX442280, JX442281, JX442282, JX442278, and JX442279, respectively.

To determine whether humans were infected with WNV, we obtained acute-phase serum samples within 1–7 days of onset of illness from persons visiting an outpatient clinic in Kashi during June 11–August 25, 2011. All patients had fever (37°C–39°C) or viral encephalitis with or without symptoms of encephalitis. Serum samples were obtained from 254 patients with fever of unknown origin and 9 patients with encephalitis.

All acute-phase serum samples were initially screened for IgM against WNV (WNV IgM Capture DxSelect; Focus Diagnostics Inc., Cypress, CA, USA) and against Japanese encephalitis virus (JEV) (JEV IgM Capture ELISA Kit; Panbio, Sinnamon Park, Queensland, Australia). A total of 38 patients (2 with viral encephalitis and 36 with fever of unknown etiology) had IgM against WNV. All samples were negative for WNV and JEV RNA.

A total of 23/38 patients positive for IgM against WNV provided convalescent-phase serum samples (obtained 18–83 days after acute-phase serum samples were obtained). All 23 paired serum samples were tested by using a 90% plaque reduction neutralization test and the XJ11029–3 strain of WNV and the P3 strain of JEV. Of these 23 serum samples, 11 had a 4-fold increase in titer of WNV-neutralizing antibody; neutralizing antibody against JEV was not detected.

Among the 11 patients who showed seroconversion, 9 had neutralization antibodies against WNV (titers 1:10 for acute-phase samples and 1:40 for convalescent-phase samples). One patient with encephalitis had a WNV antibody titer of 1:10 for an acute-phase sample and 1:160 for a convalescent-phase sample. Another patient with encephalitis had a WNV antibody titer of 1:640 for an acute-phase sample and of 1:5,120 for a convalescent-phase sample. All 11 case-patients were reported during July 28–August 23, 2011.

This study and other reports of fever and human encephalitis caused by WNV in Xinjiang, China, in 2004 (*8*,*9*) suggest that infections with WNV might be greatly underestimated. In addition, although JEV is present in this region and WNV has not been isolated in China, some patients might have been given misdiagnoses of infection with JEV because of cross-reactivity between these 2 viruses (*10*). Therefore, nationwide surveillance programs for WNV in China are needed.
